# Process Control in Jet Electrochemical Machining of Stainless Steel through Inline Metrology of Current Density

**DOI:** 10.3390/mi10040261

**Published:** 2019-04-18

**Authors:** Matin Yahyavi Zanjani, Matthias Hackert-Oschätzchen, André Martin, Gunnar Meichsner, Jan Edelmann, Andreas Schubert

**Affiliations:** 1 Professorship Micromanufacturing Technology, Faculty of Mechanical Engineering, Chemnitz University of Technology, 09107 Chemnitz, Germany; matthias.hackert@mb.tu-chemnitz.de (M.H.-O.); andre.martin@mb.tu-chemnitz.de (A.M.); andreas.schubert@mb.tu-chemnitz.de (A.S.); 2 Fraunhofer Institute for Machine Tools and Forming Technology, 09126 Chemnitz, Germany; Gunnar.Meichsner@iwu.fraunhofer.de (G.M.); Jan.Edelmann@iwu.fraunhofer.de (J.E.)

**Keywords:** electrochemical machining (ECM), process control, current monitoring, current density, surface roughness, inline metrology

## Abstract

Jet electrochemical machining (Jet-ECM) is a flexible method for machining complex microstructures in high-strength and hard-to-machine materials. Contrary to mechanical machining, in Jet-ECM there is no mechanical contact between tool and workpiece. This enables Jet-ECM, like other electrochemical machining processes, to realize surface layers free of mechanical residual stresses, cracks, and thermal distortions. Besides, it causes no burrs and offers long tool life. This paper presents selected features of Jet-ECM, with special focus on the analysis of the current density during the machining of single grooves in stainless steel EN 1.4301. Especially, the development of the current density resulting from machining grooves intersecting previous machining steps was monitored in order to derive systematic influences. The resulting removal geometry is analyzed by measuring the depth and the roughness of the machined grooves. The correlation between the measured product features and the monitored current density is investigated. This correlation shows that grooves with the desired depth and surface roughness can be machined by controlling current density through the adjustment of process parameters. On the other hand, current density is sensitive to the changes of working gap. As a consequence of the changes of workpiece form and size for the grooves intersecting premachined grooves as well as the grooves with a lateral gap, working gap, and current density change. By analyzing monitoring data and removal geometry results, the suitability of current density inline monitoring to enable process control is shown, especially with regards to manufacture products that should comply with tight predefined specifications.

## 1. Introduction

Jet-ECM, like other electrochemical machining processes, is based on anodic dissolution of workpiece material. In electrochemical machining, the machined material is influenced neither thermally nor mechanically by the removal process. Hence, complex microstructures can be machined with high precision regardless of the mechanical properties like hardness and ductility of workpiece material. Due to the mentioned characteristics ECM has become an alternative process to conventional and other nonconventional machining processes. In most of the established electrochemical machining processes the flexibility is restricted, since the shape of removal geometry is defined by the shape of cathode. In contrast, Jet-ECM is a shape generating technique, where the motion strategy defines the shape of machined part. Hence, no complicated cathode geometries are required for machining complex structures [1,2,3].

**Figure 1:**
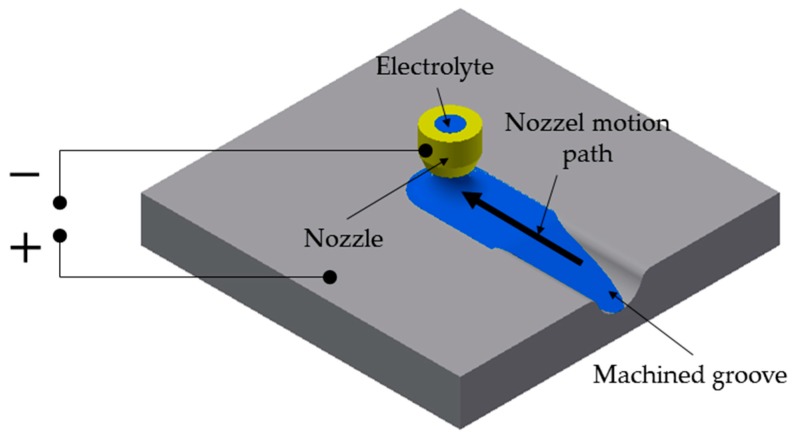
Scheme of Jet electrochemical machining (Jet-ECM).

One of the special characteristics of Jet-ECM is the application of an electrolyte jet ejected from a micronozzle. The electrolyte is ejected perpendicularly towards the workpiece surface surrounded by atmospheric air that forms a closed free jet. Microstructures can be machined by controlling the multidimensional motion of the nozzle [1]. The creation of microstructures can be controlled by switching the applied electric potential [4] or by controlling the gap between nozzle and workpiece surface [5]. In a recent research study, the effects which occur as a result of the variation of the incident jet angle were investigated [6]. The basic principle of Jet-ECM is shown in Figure 1.

**Figure 2:**
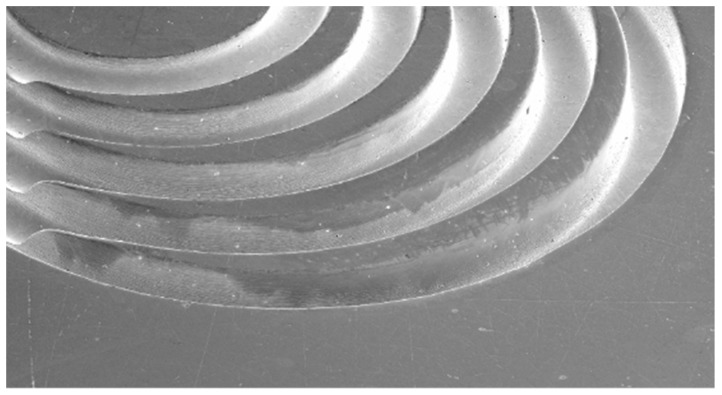
Jet-ECM of sharp edges in steel 1.4541, nozzle motion path from top right to left.

Because of the specific technical arrangement, different machining tasks can be realized by Jet-ECM, such as microdrilling [3,7,8], cutting [3], or turning [9,10]. In microdrilling, bores with vertical walls can be generated by applying Jet-ECM with a trepanning movement of the nozzle [3]. Jet-ECM is also capable of realizing sharp edges [2]. Figure 2 shows five coaxial circles with varying radii from 1 mm to 2.2 mm and different numbers of crossings from 10 to 50, which has led to a minimal edge radius of ~1 µm. This makes Jet-ECM a potential machining process to produce sharp edges. 

Furthermore, the suitability of Jet-ECM as a finishing process was investigated. Kawanaka et al. investigated the influences of current conditions and nozzle movement speed on surface roughness on stainless steel (SUS304) and realized a mirror-like surface with *Rz* surface values less than 0.2 µm. They have shown that surface roughness of grooves decreases with the increase of nozzle translating speed, and for a constant speed, the surface roughness of the grooves decreases and, after reaching a minimum value, increases again [11]. Moreover by help of inverse Jet-ECM, finish-machining of micro bores was demonstrated in simulations and experiments [12,13]. 

In Jet-ECM processes, the working gap is one important process parameter, which affects other process parameters among which current and, consequently, current density are of high importance. Schubert et al. have shown that by increasing the working gap, current and current density decrease and result in lower removal rates as well as different surface roughness, while the depth of cavities changes significantly with the working gap and no remarkable changes of width were seen [4]. They have shown that by applying 35 V, a 30% NaNO_3_ solution as electrolyte, nozzle diameter *d_n_* = 100 µm, and nozzle speed of 500 µm/s, mean current density decreases from 2100 A/cm^2^ for working gap *a* = 5 µm to 400 A/cm^2^ for *a* = 100 µm for machining of EN 1.4541 [4]. In order to control the working gap, different strategies based on electrostatic probing before machining have already been studied. When the normal vector of the surface is calculated based on touching three points on the surface of workpiece to adjust working gap, the strategy is called “adjusting by normal vector”. In the “adjusting by grid” strategy, which is used for more complicated shape deviations, multiple points can be detected and the normal vectors of the corresponding areas can be calculated from the determined values. “Adjusting by reference points” is another strategy where an individual number of points along the removal geometry are detected. Besides, the working gap can also be controlled dynamically during the process where it is determined and adjusted when differing from a defined tolerance. This strategy is called “control dynamical” [5,14]. The precision of gap measurement increases when more points are detected. However, the measurement time becomes longer as well.

The applied voltage is considered as another important process parameter where higher voltages result in considerable higher currents and current densities consequently, especially with smaller working gaps less than 50 µm and *d_n_* of 100 µm [4]. Several other process parameters, such as nozzle motion speed, nozzle diameter, electrolyte flow velocity, and electrolyte concentration, have significant influences on the machining result. The electrolyte flow is very important to ensure the complete removal of heat and gases produced by the reactions at either electrode and to let current flow to enable charge transport [15]. The nozzle diameter influences the distribution of current density and resulted removal geometry consequently. According to Schubert et al., it is shown that the depth of cut increases with nozzle diameter from 29 µm for *d_n_* = 60 µm to 77 µm for *d_n_* = 200 µm [16]. The type and concentration of salt in the electrolyte are chosen depending on the material and the need to provide sufficient conductivity to assure the dissolution of the workpiece material with adequate removal rate [17]. Table 1 shows the values of applied process parameters for different materials and the achieved results. Electrolyte type is usually selected based on workpiece material. According to the table, NaCl solutions are mostly used in Jet-ECM as a nonpassive electrolyte and NaNO_3_ with the mass concentration of 20% to 30% as a passive electrolyte. Besides, nozzles diameters range from 100 µm to 510 µm; working gaps are also in the same range. Common potential values for Jet-ECM is up to 60 V, and nozzle speeds amounting to 1000 µm/s have been studied. These parameters result in up to 250 µm of depth and the Sa roughness of less than 1.5 µm. In this study, the used parameters were chosen with regards to Table 1.

Although several researches have already been done in the field of inline metrology, additional research efforts especially in Jet-ECM are still required to control this process. Thus, the objective of the present study is to focus on inline metrology of current density as a powerful method to control process. For this purpose, systematic experiments have been carried out to monitor the development of electric current during the machining of single and intersecting grooves. The measured data were used to calculate the average current density. The influence of different voltage levels as well as different working gaps on the resulting current densities and the removal geometries in machining singles grooves are shown. Furthermore, the influence of intersecting grooves on the resulting current density was analyzed. It is shown that changes in current density affect geometrical features such as the removal depth and surface roughness of the machined grooves. In order to highlight the importance of current density monitoring, the changes of the mentioned features in dependence of changes in current density were analyzed.

## 2. Materials and Methods

Working gap and machining voltage are considered as main process parameters in Jet-ECM, since these parameters mainly influence the machining current and the current density consequently. The changes of these parameters lead to the variations of resulting removal geometries.

The mathematical basis for the calculation of the anodic material dissolution is described by Faraday’s law of electrolysis. As a result of its reversal, the material removal in the form of mass m is determined quantitatively according to Equation (1) [23].

(1) $$m=\frac{M}{z\cdot F}\cdot Q$$

Here, *M* is the molar mass of the dissolved material and *Q* is the electric charge that has been exchanged during the ECM process. The removal mass at the anode is therefore proportional to the molar mass and the exchanged electric charge [23]. F is the Faraday constant (F = 9.64853 × 10^4^ C/mol) and *z* the electrochemical valence of an ion of the ablated material. The calculation of the electric charge *Q* results from the integration of the time-dependent electric current *I(t)* over the processing time *t*. Mathematically, this relationship is described according to Equation (2) [24] where $t_{1}$ and $t_{2}$ are the times correspond to the start and the end of machining process.

(2) $$Q=\overset{t_{2}}{\underset{t_{1}}{\int }}I\left(t\right)\;dt$$

Taking into account the density ρ of the removed material, the dissolved material volume *V* is calculated by extending Equation (1) according to Equation (3) [3].

(3) $$V=\frac{M}{\rho \cdot z\cdot F}\cdot Q$$

As can be seen, the machinability of a material does not depend on its mechanical properties, like hardness or toughness, and is only characterized by its electrochemical properties. This makes EC machining an alternative technique, especially for hard-to-machine materials [23]. By a simplification assuming that *z* = *const.*, *V_sp_* represents a material constant, which is calculated according to Equation (4) [21].

(4) $$V_{sp}=\frac{M}{\rho \cdot z\cdot F}$$

Faraday’s law presupposes that all the electrical charge *Q* exchanged during the process is consumed for material removal. However, this can only be achieved under idealized conditions and cannot be attained in practice. A part of the electrical energy is consumed at the electrodes for ablation-ineffective reactions, such as the formation of hydrogen or oxygen. In addition, the formation of an oxide layer on the workpiece surface, which is depending on the interaction of the electrolyte with the workpiece material, influences the removal process and significantly reduces the efficiency of the anodic metal dissolution [25]. 

In order to take this into account, the current efficiency *η* is used for determining the efficiency of the removal process. The current efficiency *η* is defined as the quotient of the effective dissolution volume *V_eff_ = V/Q* and the specific dissolution volume *V_sp_*, according to Equation (5) [3].

(5) $$\eta =\frac{V_{eff}}{V_{sp}}$$

The current efficiency is not limited to a maximum of 100%, since it is mainly influenced by the determination of the electrochemical valence *z*, thus the current efficiency can be reasonably discussed only under consideration of *z* used for its calculation [26]. A variety of research topics includes the quantitative determination of z using micro flow cells or precision weighing [27,28,29,30,31]. Consequently, the electrochemical valence cannot be taken as an integer constant, but rather needs to be considered as a real number depending on the local electrical current density *J*. 

The electric current density is thus a decisive process variable, which is calculated according to Equation (6) as a function of the local electric current *I* (consequently applied voltage and the electrical resistance according to Ohm’s law) and the electrode surface *A_E_* [23].

(6) $$J=\frac{U}{R\cdot A_{E}}$$

The electrical resistance results from the working gap a between the tool and the workpiece and from the specific electrical conductivity κ of the electrolyte solution [23] according to Equation (7).

(7) $$R=\frac{a}{\kappa \cdot A_{E}}$$

The electrical resistance changes during the process due to the anodic removal of workpiece material and the associated enlargement of the working gap as well as the pollution and temperature-related change in electrical conductivity of the electrolyte. Hence, the working gap and the machining voltage influence the current density and thus the EC removal. This highlights the importance of current monitoring for the development of an adequate process control. In the following paragraphs, the experimental analysis of the effect of these parameters on the product features will be discussed.

## 3. Experimental Setup

**Figure 3:**
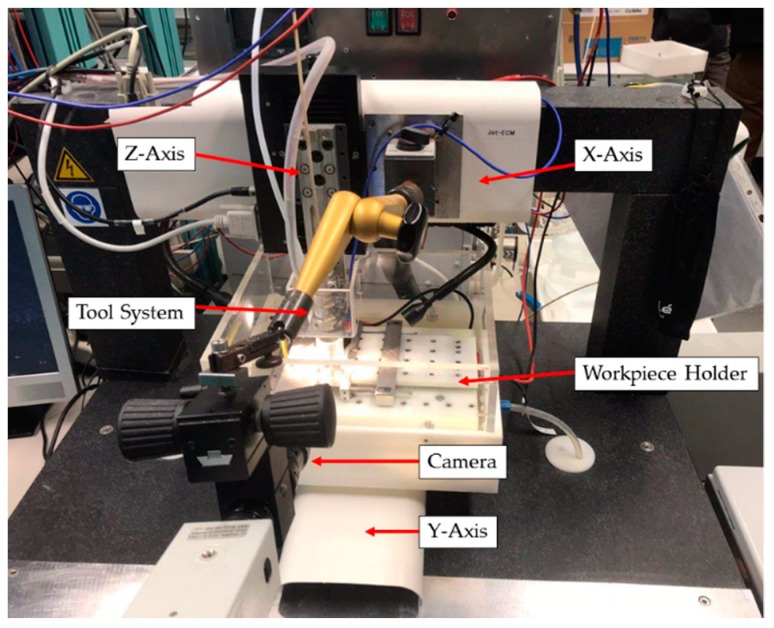
Photo of the applied Jet-ECM prototype system.

Figure 3 shows the applied in-house built Jet-ECM prototype system, which is composed of a table and a portal made of granite to guarantee the required mechanical and thermal stiffness. The relative movement between nozzle and workpiece is carried out by a linear three-axis positioning system. A pulsation-free pump transports the electrolyte to the nozzle. The electrolyte is ejected in Z direction towards the workpiece. The spent electrolyte is collected in a disposal tank. A process energy source supplies the electric voltage between the nozzle and the workpiece providing the required process current. A personal computer serves as a control system for all electrical and kinematic operations of the system [32].

The current measurement was realized using a Keysight 34465A (Keysight Technologies, Santa Rosa, CA, USA) digital multimeter. For communication with the multimeter a custom control software was developed, based on National Instruments LabVIEW (14.0), in order to measure the required data and to visualize the time-dependent development during the process. The measured data are saved to text files in order to be used for analyses after the machining processes.

### Design of Experiments

For the evaluation of the current measurement three sets of experiments were designed: (1) a set of single grooves were machined with different machining voltages and working gaps to analyze the influence of these parameters on the resulting current density, (2) a grid of single grooves to evaluate intersection characteristics, and (3) a set of parallel grooves with varying lateral distance between the grooves. The second and the third sets were executed to investigate the effects of previously machined grooves on the current density and resulting removal geometry of the postmachined grooves. 

The process parameters of the above mentioned experiments are charted in Table 2.

**Figure 4:**
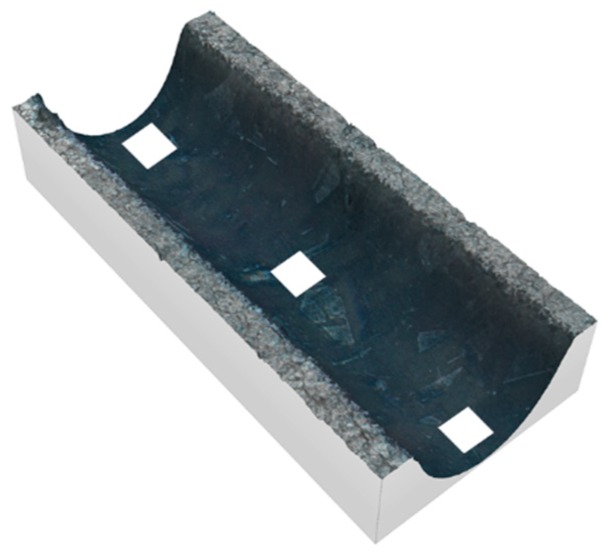
3D image of a single groove with roughness measurement areas.

Various values of working gap and machining voltage were selected based on past experience to reach a wide range of current density which, as expected, resulted in the variations of product features of the machined single grooves. For the evaluation of product fingerprints, the depth *d* and the surface roughness *Sa* of the grooves were measured by a Keyence VK-9700 (Keyence Corporation, Osaka, Japan) confocal microscope. For single grooves, three or more areas of the groove were selected (as shown in Figure 4) and the roughness in the grooves bottom were measured. The average depth and roughness were calculated and used to evaluate the relation between the mean current density and the product features.

**Figure 5:**
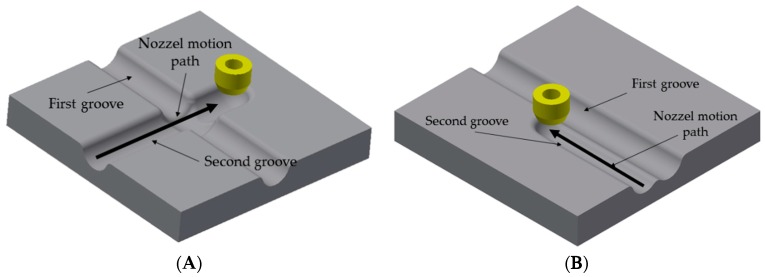
Schematics of machining directions for (**A**) intersecting and (**B**) parallel grooves, the electrolyte is not shown.

For investigations on the influence of premachined grooves on the subsequently machined grooves crossing each other rectangularly, a grid of grooves in the direction of X and Y was machined. The electric current was measured during machining the second grooves with special focus on deviations when the nozzle crossed the premachined groove. In order to investigate the influence of parallel grooves on each other, a set of parallel grooves with the machining voltages of 60 V (for both grooves) and the previously mentioned process parameters were machined. The lateral distance between the two parallel grooves was increased from 10 to 200 µm. The resulted data from current monitoring and depth measurement was analyzed to characterize the influence of the degree of superposition on the resulted mean current and removal depth of the subsequent grooves. The schematics of machining directions for intersecting and parallel grooves are shown in are shown in Figure 5A,B, respectively.

## 4. Results

### 4.1. Single Grooves

**Figure 6:**
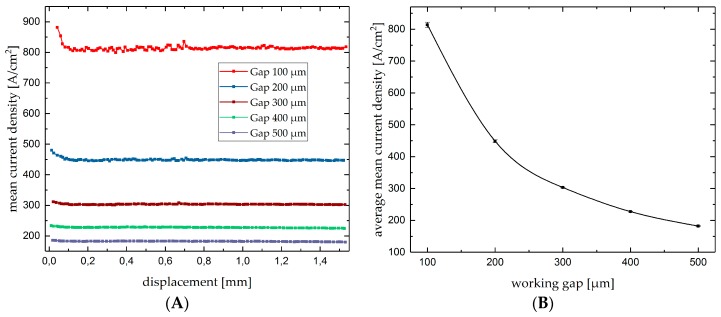
Mean current density as a function of the nozzle displacement (**A**) and average mean current density as a function of working gap for U = 60 V (**B**).

After machining the measured current data was used to analyze the current density development for machining single grooves. The current values were divided by the inner area of the nozzle to calculate the plotted mean current density *J_m_* [11]. As an example, the current density developed during the machining of single grooves with the machining voltage of 60 V as function of the nozzle displacement for the analyzed working gaps is shown as point diagram in Figure 6A. It can be seen in Figure 6A that no major changes in the current density were seen during machining single grooves over a plane workpiece surface.

Figure 6B shows the average mean current density as a function of the working gap for 60 V. The error bars indicate the standard deviations calculated from the single measurement values of the Figure 6A. As can be realized, the standard deviations are comparatively low (<1%) for all working gaps. Hence, machining of single grooves on a plane workpiece surface only leads to slight deviations in mean current density, but significant changes in mean current density were detected due to changes in working gap while the voltage was kept constant. This indicates that mean current density is a proper process parameter for the control of the working gap during Jet-EC milling of single grooves. Besides, as shown in Table 2, apart from different working gap sizes, varying machining voltages were also applied. The variation of working gap as well as machining voltage lead to different values of current density.

**Figure 7:**
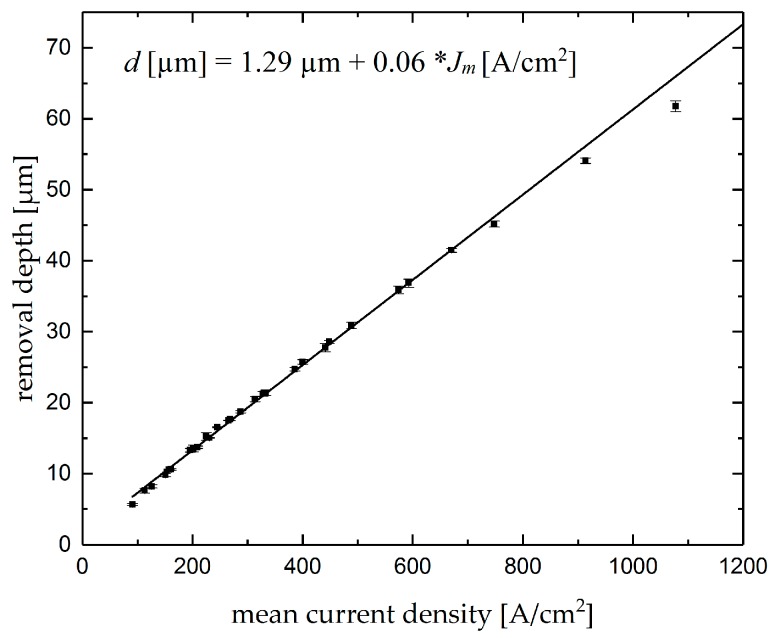
Removal depth of single grooves as function of the mean current density.

Figure 7 shows the removal depth as a function of the current density in machining single grooves. The solid line shows the linear fit of the points. As can be seen in the graph, the depth of the groove increases linearly with increasing mean current density. The linear function between the mean current density and the depth of the single grooves underlines that controlling the mean current density is a useful tool for targeted machining of single grooves with predefined removal depth. This correlation has been stated in mathematical form in Equation (8).

*d* [µm] = 1.29 µm + 0.06 × *J_m_* [A/cm^2^] (8)

**Figure 8:**
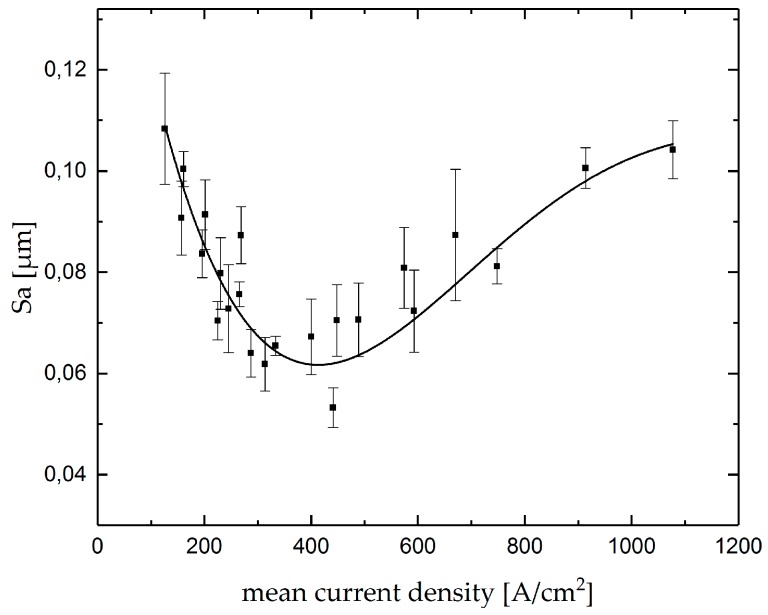
Aerial roughness Sa as function of the mean current density in Jet-EC milling of single grooves.

As an important product feature, the aerial roughness value *Sa* of the single grooves was measured. The result of the roughness measurements as a function of the mean current density is shown in Figure 8. The point diagram shows a decrease in roughness with an increase in mean current density up to a value of approximately 400 A/cm^2^, while a further increase in mean current density results in an increasing roughness. An adequate control of the mean current density offers the possibility for finish-machining in order to achieve a predefined surface roughness *Sa*.

### 4.2. Intersecting Grooves

**Figure 9:**
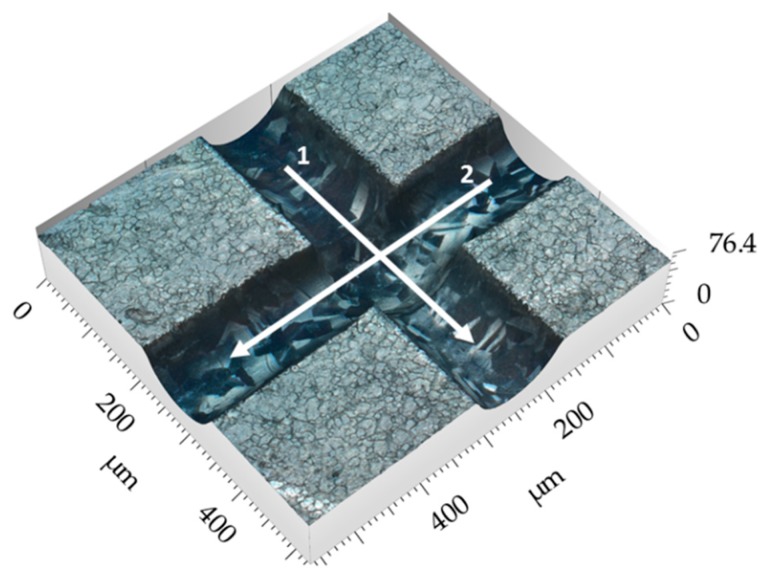
3D image of intersecting grooves.

Figure 9 shows a 3D image of the intersection between two Jet-EC milled grooves, where both grooves were machined with 60 V.

The measured depths of the premachined grooves are shown in Table 3. 

**Figure 10:**
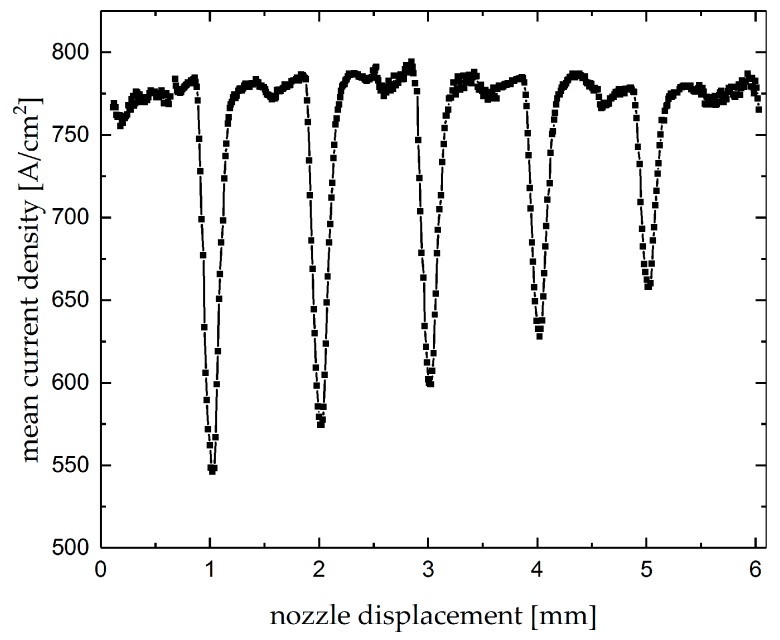
Mean current density as function of the nozzle displacement during Jet-EC milling of subsequent grooves with a voltage of 60 V crossing premachined grooves with different depths.

Figure 10 shows a point diagram of the mean current density as function of the nozzle’s displacement during machining a subsequent groove and crossing these five premachined grooves rectangularly. The premachined grooves were machined with a lateral distance of 1 mm from each other. As can be seen, the mean current density drops significantly when the nozzle crosses the premachined grooves, which indicates the sensitivity of the electric current to local changes of the working gap due to the deviations of workpiece surface.

**Figure 11:**
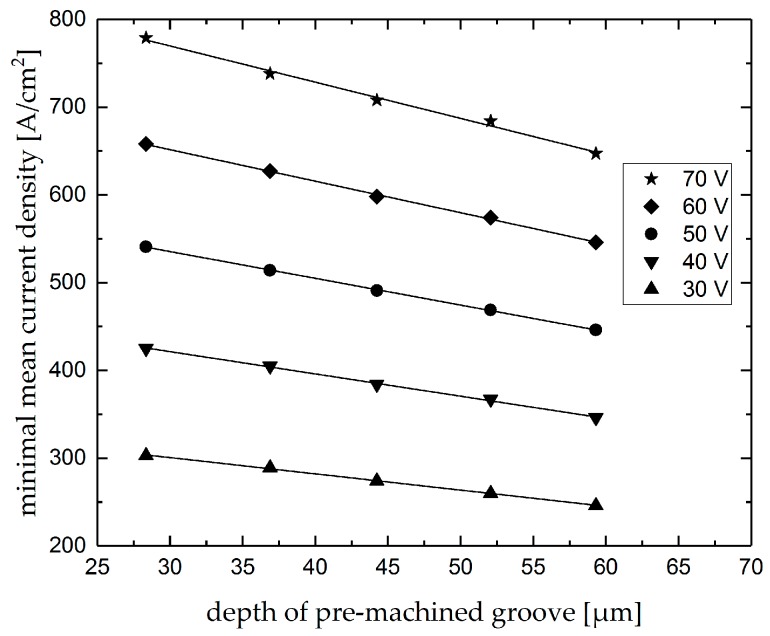
Minimum mean current densities of intersections as a function of the depth of premachined grooves for differing voltages.

In Figure 11, the measured minimum mean current density *J_min_* of the five intersecting positions are displayed as a function of the depth of the premachined groove for all the analyzed voltages. The point diagram shows that the minimum mean current density decreases linearly with increasing depth of the premachined groove. Hence, the minimum mean current density can be considered as an indicator for the value of surface deviations depending on the removal depth of the premachined grooves. As can be seen in Figure 11, the linearity of changes of the minimum current density with depth of premachined grooves is independent of machining voltage and therefore, with low or high machining voltages, the amount of surface deviations can be characterized.

**Figure 12:**
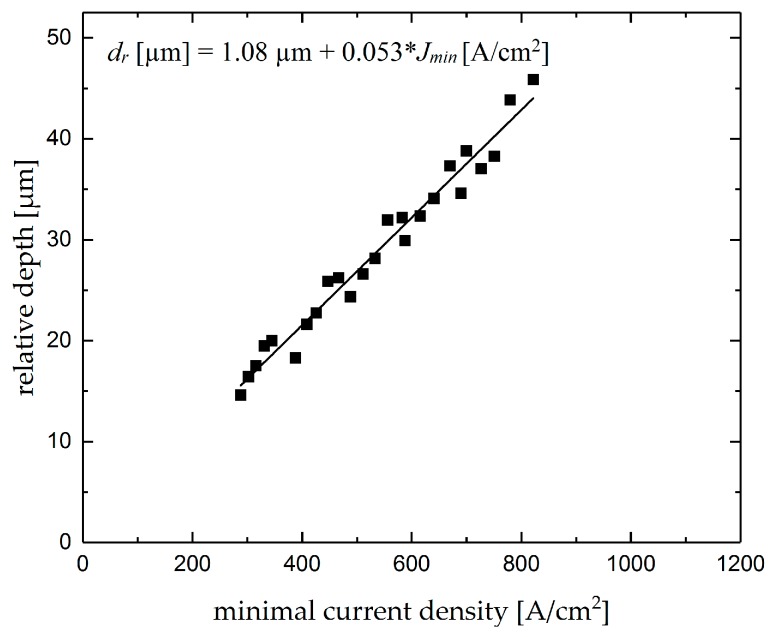
Changes of intersection relative depth with minimum current density.

For the intersecting grooves the measurements of the surface roughness and the removal depth of the intersections were carried out with the mentioned confocal measurement system. After preliminary investigations, including the evaluation of minimum and average current density over the intersections, it was found that the minimum current density occurs when the nozzle crosses the center of the intersections, which can be characterized as a specific product features. As another specific product feature, the relative depth *d_r_* was calculated from the difference between the maximum depth in the intersection and the depth of the premachined groove. Figure 12 shows the calculated relative depths as function of the minimum current density. 

Similar to the analyzed removal depth as a function of the mean current density in machining single grooves, the relative depth of the intersecting grooves increases linearly with an increase in minimum current density, as stated in Equation (9). In order to ease the comparison with the single grooves, the same scale was used for this graph. The data of this graph together with the results of Figure 11 can enhance the current monitoring with online control of the first groove depth as well as the intersection depth while the minimum value of current density over an intersection is proportional to the first groove depth, and this value can be used to estimate the relative depth of the intersection.

*d_r_* [µm] = 1.08 µm + 0.053 × *J_min_* [A/cm^2^] (9)

**Figure 13:**
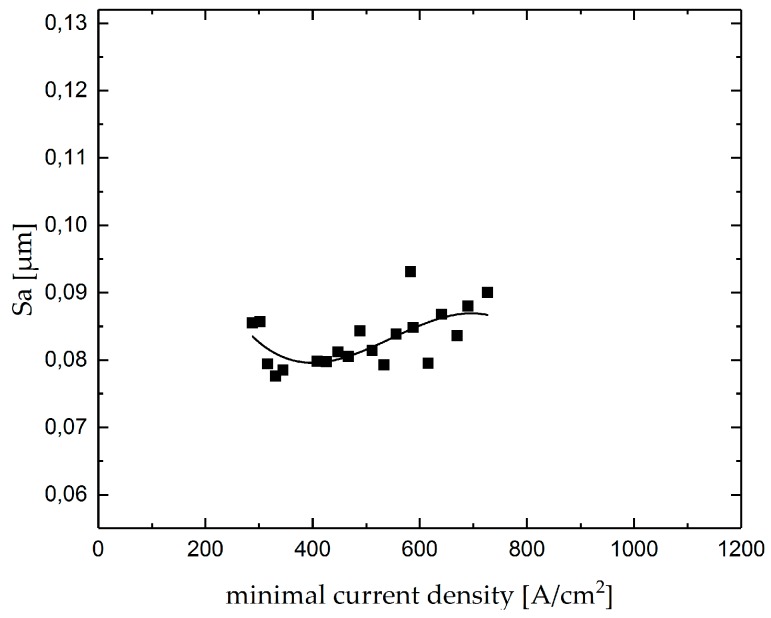
Aerial roughness (Sa) as function of the minimal current density in Jet-EC milling of intersecting grooves measured in the center of the intersections.

Similar to the results in machining single grooves, the surface roughness of intersecting grooves decreases with increasing minimum current density up to a value of approximately 400 A/cm^2^ and increases again at further increase in minimum current density, although the slope of changes is less significant than the slope determined for single grooves as can be seen in Figure 13.

### 4.3. Parallel Grooves

**Figure 14:**
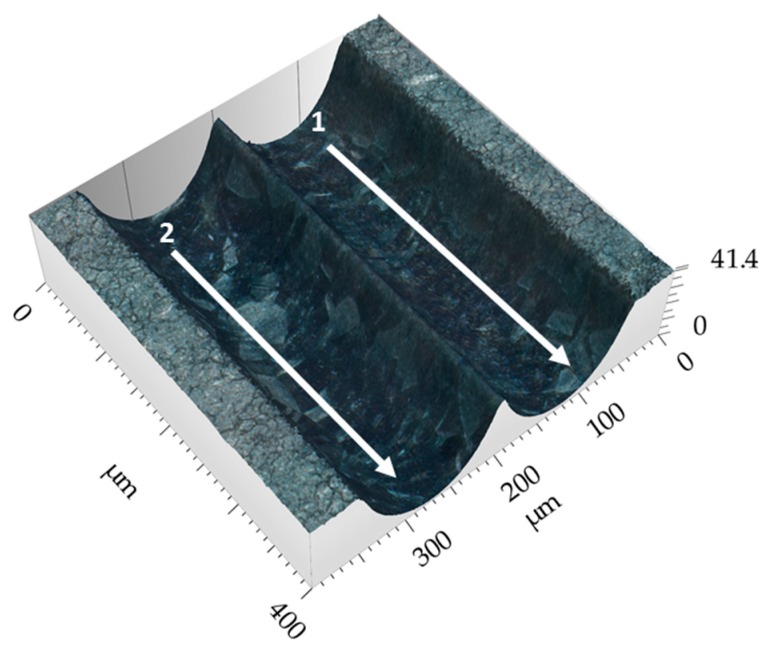
3D image of parallel grooves.

Figure 14 shows a 3D image of parallel grooves with the lateral distance of 150 µm.

**Figure 15:**
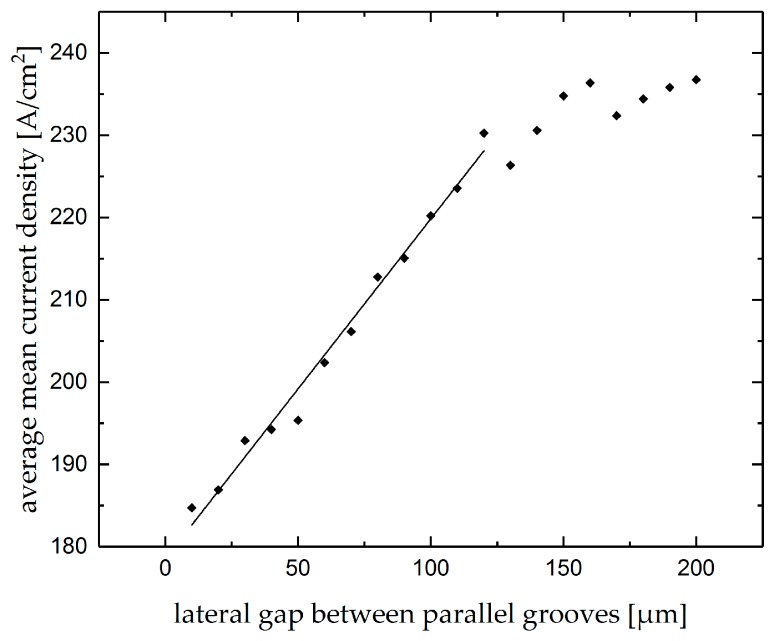
Average mean current density of the subsequent grooves as function of the lateral gap from the premachined parallel groove.

The average means current density measured during machining subsequent groove as function of the lateral gap from the premachined parallel groove is shown in Figure 15. As can be seen, the average mean current density increases linearly with increasing lateral gap between the two parallel grooves in a range from 5 µm to 120 µm. Between 120 and 160 µm, the average mean current density rises slightly, and the influences of lateral gap are hardly detected for the gaps wider than 160 µm.

**Figure 16:**
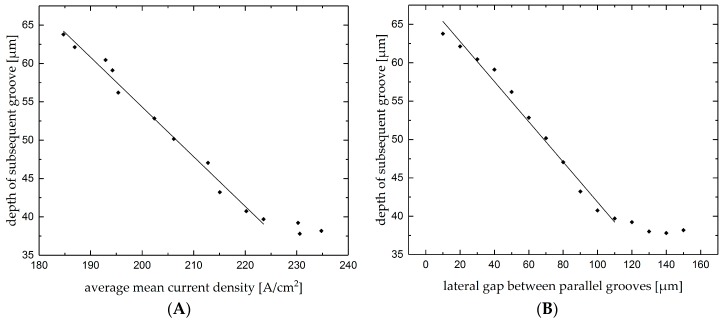
Depth of second parallel groove as function of (**A**) mean current density and (**B**) lateral gap.

As a specific product feature in this case the depth of the subsequent groove was measured and characterized according to influences of the premachined groove. In Figure 16A, the changes of the depth of the subsequent groove with average mean current density is plotted. As can be seen, the depth of the subsequent groove decreases linearly at increasing average mean current density up to a value of 225 A/cm^2^. This can be explained by the changes of current density distribution where by the increase of lateral gap, the actual working gap decreases and more material is ablated from the side of the groove rather than the bottom. According to Figure 15 this corresponds to the value at a lateral gap of approximately 120 µm, up to which the premachined groove affects the average mean current density when machining the subsequent groove. Hence, at a further increase in average mean current density only slight changes were detected. 

Figure 16B shows the depth of the subsequent groove as function of the lateral gap between the grooves. The depth of the subsequent groove decreases linearly with increasing lateral gap up to a value of approximately 120 µm, which corresponds to the results asserted in Figure 15 and Figure 16A, where little influence of the premachined groove on the subsequent groove was detected at wider lateral gaps. The results indicate that the control of the current density is a useful tool for targeted machining of parallel grooves with predefined removal depth and in specific ranges, when the lateral gap is smaller than the nozzle diameter, can be used to measure the actual lateral gap of the parallel grooves.

**Figure 17:**
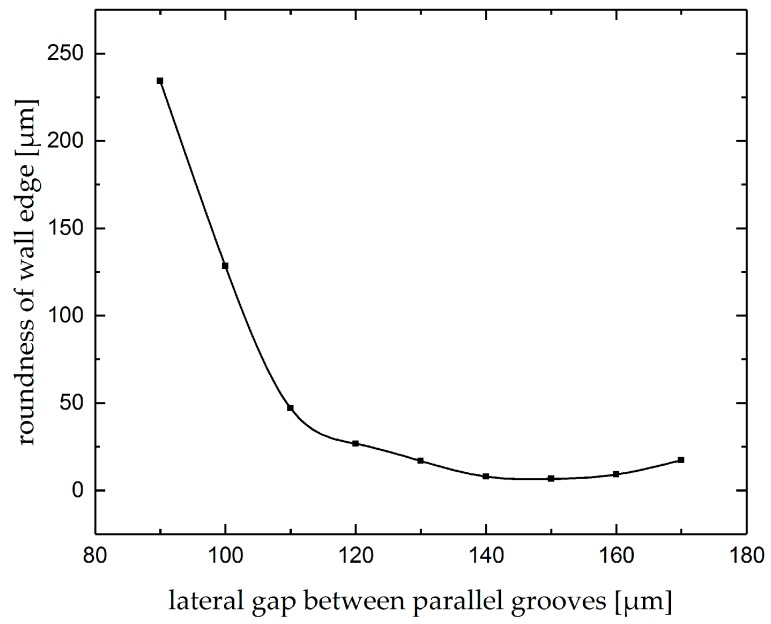
Roundness of the edge of thin wall between parallel grooves.

As another feature of the product, the roundness of the edges of the walls between grooves was investigated. Figure 17 shows the variation of this feature as a function of lateral gap. For lateral gaps smaller than 90 µm, the edge cannot be detected. As can be realized, the roundness of the edge decreases significantly up by increasing the gap up to 120 µm. The roundness of the edge changes slightly with the lateral gaps between 120 and 160 µm. As discussed before, the mean current density of the lateral groove increases slightly in this range of lateral gap. For lateral gaps bigger than 160 µm, no influence of the first groove was seen.

## 5. Conclusions

This paper has highlighted the importance of current density measurements in Jet-ECM process control. The results indicate that current density is very sensitive to the changes of working gap. For single grooves, provided there is a constant machining voltage, current density changes significantly by changing the preset working gap. On the other hand, product features of complex microstructures can be monitored during the process by measurement of current density.

The results of the analyses can be summarized as below.

For single grooves

- depth changes linearly with current density and
- surface roughness decreases with the increase in current density and then increases again.

Considering the above, in order to reach desired depth and roughness, a combination of process parameters which lead to specific current density should be selected. On the other hand, current monitoring during machining can be used to predict the product features before further measurements.

For intersecting and parallel grooves

- minimum current density over intersections changes proportionally to the depth of premachined grooves for each machining voltage level, which can be used as a monitoring tool for the first groove depth;
- the relative depth of intersections showed linear changes with the minimum current density over the intersection. Therefore, minimum depth over intersection can be applied for the prediction of the relative depth; and
- the depth and the mean current density of subsequent parallel grooves changes linearly with the lateral gap. This enhance the process monitoring with useful data of the actual lateral gap as well as the depth by monitoring the mean current density.

These results in an initial step toward Jet-ECM process control. In further research, experiments will be done to analyze the current density over different micro features on the workpiece.

## Figures and Tables

**Table 1:** Applied process parameters and achieved results in previous studies for different materials.

Parameter	Value									
Workpiece Material	Co [18]	WC [18]	WC- 6% Co [18]	Nimonic 80A [19]	Ti-6Al-4V [20]	EN 1.2379 [21]	EN 1.4301 [21]	EN 1.4541 [21]	EN 1.5920 [21]	Brass, Cu39Zn2Pb [22]
Nozzle inner diameter (µm)	100	100	100	100	250	100				510
Electrolyte	20% NaCl	20% NaCl	20% NaCl	20% NaCl	2–4 M NaNO_3_	30% NaNO_3_				2.3 M NaNO_3_
Working gap (µm)	100	100	100	100	500	100				500
Voltage (V)	50	50	10–55	1–56	-	56				-
Nozzle speed (µm/s)	200	200	200	150	0 Machining time: 10s	200–1000				500
Depth of removal (µm)	40	< 1	4–5	300	50–250	75–240	60–230	60–220	100–250	150 µm/C
Surface roughness (µm)	-	-	Ra < 0.65	-	-	0.35 < Ra < 0.45	0.1 < Ra < 0.15	0.15 < Ra < 0.33	0.3 < Ra < 0.45	0.3 < Sa < 1.5

**Table 2:** Process parameters for machining single, intersecting, and parallel grooves.

Parameter		Value
Workpiece material		EN 1.4301
Nozzle inner diameter		100 µm
Electrolyte		30% NaNO_3_
Electrolyte supply rate		10 mL/min
Working gap	single grooves	100, 200, 300, 400, and 500 µm
	intersecting grooves	100 µm
	parallel grooves	100 µm
Voltage	single grooves	30, 40, 50,60, 70, 80, and 90 V
	intersecting grooves	30, 40, 50, 60, and 70 V
	parallel grooves	60 V
Nozzle speed		200 µm/s

**Table 3:** Machining voltage and depth of first grooves of intersecting groove.

Process and Geometry Parameter	Value				
Machining voltage (V)	70	60	50	40	30
Depth of groove (µm)	59	52	44	36	28
